# Remote homology and the functions of metagenomic dark matter

**DOI:** 10.3389/fgene.2015.00234

**Published:** 2015-07-21

**Authors:** Briallen Lobb, Daniel A. Kurtz, Gabriel Moreno-Hagelsieb, Andrew C. Doxey

**Affiliations:** ^1^Department of Biology, University of WaterlooWaterloo, ON, Canada; ^2^Department of Biology, Wilfrid Laurier UniversityWaterloo, ON, Canada

**Keywords:** metagenome, metaproteome, ORFan, orphan, remote homology, profile-profile comparison, functional annotation, comparative metagenomics

## Abstract

Predicted open reading frames (ORFs) that lack detectable homology to known proteins are termed ORFans. Despite their prevalence in metagenomes, the extent to which ORFans encode real proteins, the degree to which they can be annotated, and their functional contributions, remain unclear. To gain insights into these questions, we applied sensitive remote-homology detection methods to functionally analyze ORFans from soil, marine, and human gut metagenome collections. ORFans were identified, clustered into sequence families, and annotated through profile-profile comparison to proteins of known structure. We found that a considerable number of metagenomic ORFans (73,896 of 484,121, 15.3%) exhibit significant remote homology to structurally characterized proteins, providing a means for ORFan functional profiling. The extent of detected remote homology far exceeds that obtained for artificial protein families (1.4%). As expected for real genes, the predicted functions of ORFans are significantly similar to the functions of their gene neighbors (*p* < 0.001). Compared to the functional profiles predicted through standard homology searches, ORFans show biologically intriguing differences. Many ORFan-enriched functions are virus-related and tend to reflect biological processes associated with extreme sequence diversity. Each environment also possesses a large number of unique ORFan families and functions, including some known to play important community roles such as gut microbial polysaccharide digestion. Lastly, ORFans are a valuable resource for finding novel enzymes of interest, as we demonstrate through the identification of hundreds of novel ORFan metalloproteases that all possess a signature catalytic motif despite a general lack of similarity to known proteins. Our ORFan functional predictions are a valuable resource for discovering novel protein families and exploring the boundaries of protein sequence space. All remote homology predictions are available at http://doxey.uwaterloo.ca/ORFans.

## Introduction

Metagenomes are a rich resource of novel genes (Godzik, 2011) from which the metabolic and physiological activities of entire microbial communities can potentially be inferred (Handelsman, 2004). This difficult task relies largely on the accuracy of current methods for predicting function from sequence, which is challenging even for single microbial genomes (Wooley et al., 2010).

Standard homology-based annotation methods have become the most common strategy for metagenome annotation (Prakash and Taylor, 2012). Here, metagenome-derived open reading frames (ORFs) are searched using BLAST (Altschul et al., 1997), or related tools, against reference protein databases such as the NCBI non-redundant (nr) and Swissprot databases. Alternatively, reads can be scanned against databases of protein domain models such as the Conserved Domain Database (CDD) (Marchler-Bauer et al., 2014) and Pfam (Finn et al., 2014), where each protein family is represented by either position-specific scoring matrices (PSSMs) or hidden Markov models (HMMs). If functionally annotated hits in the databases are detected, functions are inherited from these hits.

Both frustrating and intriguing are the many predicted genes within metagenomes (and genomes) that cannot be readily annotated using standard homology-based methods. The most challenging among these genes are the *ORFans*, genes that lack detectable homologs in the database (Siew and Fischer, 2003). Initially identified in some of the first genomes (Dujon, 1996), ORFans have become a universal feature of newly sequenced genomes and metagenomes, despite an exponential increase in sequencing (Tautz and Domazet-Lošo, 2011). Estimates of ORFan content in metagenomes vary from 25 to 85% of total genes (Prakash and Taylor, 2012). This proportion depends on numerous factors including read length, metagenome complexity, species novelty, homology detection methods and significance thresholds. In addition, a large fraction of metagenome-derived sequences come from microorganisms that resist current cultivation techniques (Gill et al., 2006), which makes them dissimilar from database sequences and hard to annotate. Prakash and Taylor (2012) showed that, of the genes in the human gut microbiome, 75% could be annotated, vs. only 50–55% of genes in “complex metagenomes” from soil and ocean environments. Another recent study of a large prairie soil metagenome reported that only 30–38% of predicted proteins had detectable similarity (≥60% identity) to proteins in NCBI's M5nr database (Howe et al., 2014), and this has dropped as low as 15% in some extreme cases (e.g., the cow rumen virome).

Several types of alternative, non-homology-based methods may be applicable to annotation of ORFan proteins. Genomic context methods, for instance, predict functions for uncharacterized ORFs based on functions of neighboring genes since gene neighborhoods in prokaryotes tend to possess a significant degree of functional consistency (Dandekar et al., 1998; Marcotte et al., 1999; Galperin and Koonin, 2000; Salgado et al., 2000; Yanai et al., 2002; Korbel et al., 2004). These “guilt by association” methods have previously been applied to metagenome annotation (Harrington et al., 2007; Vey and Moreno-Hagelsieb, 2010) but depend on assembled contigs, which can be difficult to obtain. Another popular class of prediction methods includes remote-homology detection approaches such as HMM profile-profile comparison. These methods are based on the principle that distant homologies may be apparent by comparison of conservation profiles between families, even if they are not apparent between single sequences (Sadreyev et al., 2003; Sánchez-Flores et al., 2008). The popular profile HMM-HMM comparison method, HHpred/HHsearch (Söding, 2005), is among the most sensitive methods for homology detection and is consistently ranked among the top automatic structure prediction methods in recent CASP (Critical Assessment of protein Structure Prediction) competitions.

To our knowledge, no studies have applied remote homology to large-scale annotation of metagenomic ORFans, perhaps due to the considerable computation required. Thus, the functions and origins of ORFans, which can be abundant in environmental sequences, are unclear. Here, we identified and analyzed ORFans from three large metagenome collections: the Great Prairie Soil Metagenome Grand Challenge (GPC), the Global Ocean Sampling (GOS), and the Human Gut Microbiome (HG), encompassing aquatic, host-associated, and terrestrial environments. Through an analysis of 35,307,707 total coding sequences (CDSs), we identified thousands of novel ORFan protein families, and inferred function for ~15% through remote homology to proteins of known structure. The structural predictions provide insights into the functions and evolutionary origins of ORFan proteins.

## Materials and methods

### Datasets and identification of metagenomic ORFans

We retrieved metagenomic sequence data from three large metagenome collections: GPC [(Howe et al., 2014); MGRAST ids 4504797.3 and 4504798.3], GOS [(Rusch et al., 2007); http://camera.crbs.ucsd.edu/projects/details.php?id=CAM_PROJ_GOS], and HG [(Qin et al., 2010); http://www.bork.embl.de/~arumugam/Qin_et_al_2010/].

For CDS prediction, FragGeneScan version 1.18 (Rho et al., 2010) was applied directly to the unassembled reads from the GOS dataset. Due to the short read lengths from the GPC and HG datasets, we applied FragGeneScan to pre-assembled metagenomes from Howe et al. (2014) and Qin et al. (2010), respectively. We used segmasker from the BLAST version 2.2.28+ package to identify repetitive regions in putative ORFs, and CDSs containing over 40% repetitive sequence were discarded. To annotate CDSs with domain family homologs, hmmsearch from HMMER version 3.1b1 was used to scan the Pfam database (Pfam-A downloaded 15 May 2014), and remaining CDSs were scanned against the Conserved Domain Database (CDD) (20 Feb. 2014 release from NCBI) using rpsblast from the BLAST version 2.2.28+ package. An *E*-value cut-off of 10^−3^ was used for both methods. CDSs without identified domain family homologs, were clustered with CD-HIT version 4.6.1 using a 60% identity threshold. Spurious CDS predictions were identified as singleton clusters (those containing one sequence), clusters whose representative (longest) sequence was shorter than 100 amino acids, and clusters comprised entirely of sequences with 99% or greater identity to the representative sequence. These spurious clusters were excluded from further analysis. Representative sequences of each remaining cluster were used for blastp database searches (downloaded 15 May 2014 from NCBI). Clusters with either no similarity to the nr database or with a top nr blast match exceeding the cutoff of *E* = 10^−3^ (used previously by Kuchibhatla et al., 2014) were defined as *ORFans*. Multiple sequence alignments of the non-spurious clusters were generated with MUSCLE version 3.8.31 (www.drive5.com/muscle), and these were further enlarged with sequences from the nr20 database (12 Aug. 2011 release from HH-suite) using HHblits from the HH-suite version 2.0.16 package with default settings.

### Remote homology detection and FDR estimation

Profile-profile comparisons were performed using HHsearch from the HH-suite version 2.0.16 package with the PDB70 HMM database (17 May 2014 release from HH-suite) and default settings. For each prediction, an *E*-value and probability score were collected. To determine appropriate thresholds, we repeated remote homolog detection using random, reshuffled alignments as described below. Based on the results, a probability threshold of 80% was chosen with the *E*-value set at 1, equivalent to a ~9% false discovery rate (see Results). To obtain an FDR estimate, the pipeline was repeated using shuffled alignments which represent artificial sequence families that maintain compositional characteristics and column-specific conservation (Margulies and Birney, 2008; Guturu et al., 2013). One thousand ORFan clusters obtained by CD-HIT were randomly selected from each metagenome, and the columns of each cluster's multiple sequence alignment were shuffled. The shuffled alignments were run through the HHblits and HHsearch algorithms as described previously using the non-shuffled clusters.

### Genomic context analysis

The CDS locations on contigs (for GPC and HG) and reads (for GOS) were used to define genomic neighbors and perform genomic context analysis. The Pfam-GO mapping from InterPro (Hunter et al., 2009) was used to assign GO terms to ORFs. For Pfam domain homologs, the GO terms of all significant (*E* < 10^−3^) domain matches were included in its functional annotation. For the non-spurious CD-HIT clusters (ORFans and clusters with homologs from the NCBI nr database), a GO term collection was assigned to each cluster based on the top three significant remote homologs found by HHsearch, using the PDB-GO annotation table obtained from the EBI (http://geneontology.org/gene-associations/gene_association.goa_pdb.gz). GO terms were assigned to each CDS within the CD-HIT cluster.

For each metagenome, we then compared the list of GO terms for an ORFan against the list of GO terms associated with its directly neighboring CDSs (one on either side, in the same orientation and within 1 kb) on the same contig, and calculated the number of shared terms (S) between both sets. This value was then summed for all ORFans within a metagenome (m) to obtain an overall statistic (S_m_) reflecting the similarity between ORFans and their annotatable genomic neighbors. To estimate statistical significance, we compared S_m_ to a null distribution computed by swapping the ORFans amongst their original locations. The count was then calculated as above, shuffling ORFans only while maintaining the positions of all other CDSs. Shuffling followed by the shared GO terms summation was performed 1000 times.

### Analysis of overrepresented functions

To determine the frequency of GO terms in each metagenome, 10,000 CDSs with Pfam domain hits were randomly selected from each metagenome and run through HHblits with only one iteration and a limit of 30 sequences in the output alignment followed by HHsearch with default settings (using the databases described previously). The functional information for ORFan sequence clusters and the subset of Pfam domain hits was gathered using the most confident GO term-associated HHsearch hit (using the PDB-GO map and only assessing significant HHsearch hits). Similar to previous studies (Van Driel et al., 2006; Vazin et al., 2009), analyses were restricted to sixth level GO terms in the biological process or molecular function trees since this level was more informative (greater biological specificity) than other trimmed ontologies such as GO Slim terms. GO term levels were calculated using the “is a” relationship, with the starting terms (biological process and molecular function) being considered level one. Only the longest path from the root terms was considered. The frequency of each GO term in the Pfam and ORFan subsets and PDB70 were calculated, with zero counts converted to a pseudocount of 1 to avoid division errors. The fold change of each GO term in the ORFan sequence clusters over the Pfam domain hits subset was calculated and compared across metagenomes. *P*-values were calculated in R using the binomial test with false discovery rate adjustment (p.adjust function) as described elsewhere (Doxey et al., 2010).

### Analysis of environment-specific ORFan families

For each metagenome, we computed the proportions of the total number of ORFans matching a PDB entry as the top remote homolog. Three-dimensional scatterplot were generated with each axes representing this quantity. The binomial test was used to compute *p*-values with background probabilities based on the total counts observed in the other two metagenomes. These *p*-values were then corrected using the Bonferroni adjustment. The same procedure was repeated based on proportions of ORFans from each metagenome possessing GO terms (1769 total terms).

### ORFan metalloprotease discovery

ORFan clusters were searched for those that: (1) possessed a top remote homolog match to a PDB entry possessing “protease” or “peptidase” terms in any functional description category; (2) had a representative sequence with at least one match to a HExxH motif. ORFan CD-HIT clusters meeting both conditions were considered putative ORFan metalloproteases or metallopeptidases.

## Results

### Identification of ORFan sequences in three large metagenomes

With the goal of characterizing ORFans from diverse metagenomes, we retrieved and analyzed three large, publicly available datasets: the Great Prairie Soil Metagenome Grand Challenge (GPC), Global Ocean Sampling (GOS), and Human Gut Microbiome (HG). We selected metagenomes from diverse biomes (terrestrial, marine, host-associated) since observed differences in ORFan content and functions may be biologically relevant while commonalities may indicate general trends.

**Figure 1:**
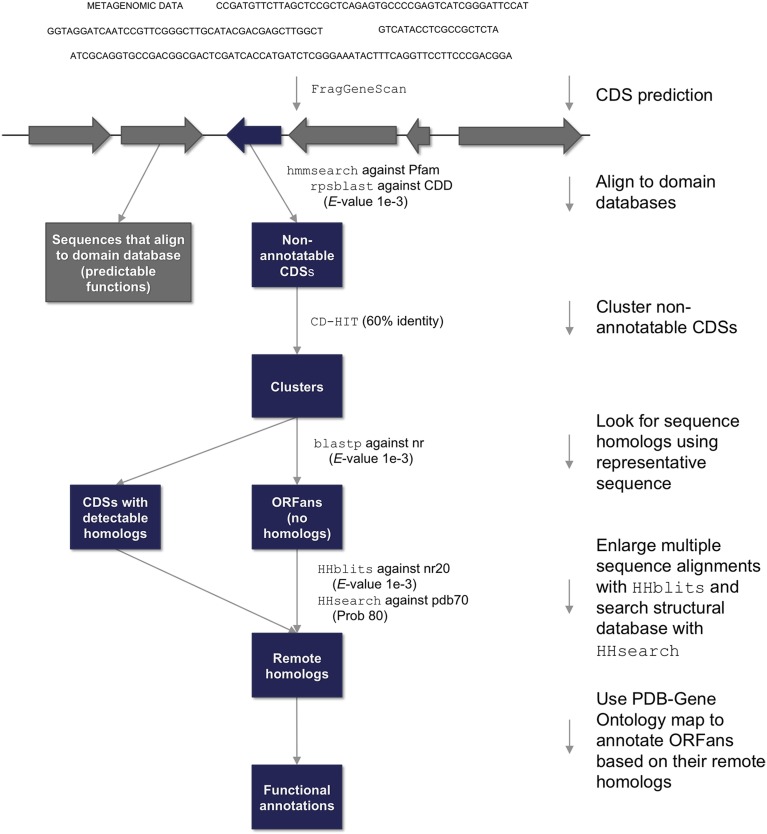
**Pipeline for detection and functional annotation of metagenomic ORFan proteins**. Protein-coding sequences (CDSs) were predicted from assembled metagenomic contigs, and searched against conserved domain databases. CDSs that could not be annotated by domain homology were further clustered, and representatives were BLASTed against the NCBI nr database. Remaining CDS clusters lacking detected homologs were considered ORFans, and these were subjected to remote homology detection using HHblits and HHsearch, which were used to perform profile-profile searches against the Protein Data Bank.

First, all genes within these metagenomes were predicted regardless of whether they could be verified through homology to known sequences. This initial set included a staggering number (35,307,707) of CDSs, equivalent to about 20% of the entries in the current NCBI GenBank database. Each CDS was processed using the computational pipeline described in Figure 1 (see Table 1 for statistics at each step), with the intention of separating the ORFans from the homology-annotatable sequences. Potential ORFans were identified as CDSs whose products lacked detectable homology to known protein domain families (Pfam and CDD) or proteins in the NCBI database (see Materials and Methods). Since these potential ORFans likely contain a mixture of real ORFan proteins and false positives (Gilbert et al., 2008), additional steps were required to remove spurious ORFs. We therefore clustered the CDSs and removed singletons (Siew et al., 2004; Gilbert et al., 2008), clusters with low sequence variation, and clusters composed exclusively of short fragments (see Materials and Methods). This left 85,422 (GPC), 251,857 (GOS), and 146,842 (HG) putative ORFan proteins from each metagenome (Table 1). By definition each ORFan within this final set is an apparent gene coding for a protein, is a member of a sequence cluster with at least one representative of 100 amino acids or longer, and yet has no detectable homology to any known protein or conserved domain family. All following analyses were performed on this set of ORFans.

**Table 1:** **Number of CDSs and ORFans at key stages of metagenomic ORFan identification**.

	**GPC**	**GOS**	**HG**
Predicted CDSs	5,606,711	17,204,095	12,496,901
CDSs removed containing conserved domain matches (Pfam + CDD)	2,480,274	4,542,071	4,674,912
Spurious (singleton, short and repetitive) CDSs removed	2,758,146	11,458,304	6,603,567
CDSs removed with BLAST matches to nr database	282,869	951,863	1,071,580
Candidate functional ORFans	85,422	251,857	146,842
ORFan CD-HIT clusters	33,013	73,428	32,078
Annotated (HHsuite) ORFan CDSs	21,358	38,900	13,638
Annotated (HHsuite) ORFan CD-HIT clusters	7848	10,973	3119

### ORFans are shorter but compositionally similar to real proteins from their environments

Next we examined whether the detected ORFans share compositional characteristics with homology-annotatable CDSs (those with PFAM or CDD domain matches) from their environments. If so, this would suggest that predicted ORFans are under similar evolutionary pressures as real proteins and indicate potential functionality. We therefore investigated the distributions of CDS length and GC content (Table 2) for each CDS category. Biases have been observed previously for ORFans (Yin and Fischer, 2006; Cortez et al., 2009; Yomtovian et al., 2010). Consistent with previous studies, ORFans tend to be shorter in all datasets (Table 2), and the relative abundance of ORFans also decreases with increasing read length (Figure S1). Overall, the GC content distributions of the homology-annotatable CDSs and ORFans are highly similar within but vary considerably between metagenomes (Figure S2). Although the length distributions are also affected by sequencing method, this is not the case for GC content, suggesting that the predicted ORFans exhibit characteristics of the *real* (homology-annotatable) CDSs from their environments.

**Table 2:** **Average G + C content and length of domain-annotated vs. ORFan sequences from three metagenomes**.

	**Average G + C content (%)**	**Average CDS length (# nucleotides, nt) excluding sequences under 300 nt**
GPC Pfam and CDD hits	56.8	411.4
GPC ORFans	54.8	407.4
GOS Pfam and CDD hits	39.2	731.7
GOS ORFans	39.4	548.7
HG Pfam and CDD hits	46.6	781.7
HG ORFans	43.0	525.2

### Many ORFans exhibit remote homology to proteins of known structure

Although ORFans, by definition, do not possess detectable homology to existing protein families using standard database search techniques like BLAST or HMMER, we were interested whether remote homology detection techniques could prove effective. We applied profile-profile, remote homology detection using HHblits/HHsearch (Söding, 2005; Remmert et al., 2011), which compares the conservation profile derived from the multiple sequence alignment (MSA) of the ORFans to those of known protein families. These methods can often identify remote relationships between protein families, even if individual members do not share detectable homology. To facilitate remote homology detection, we first generated initial MSAs for each ORFan cluster, and detected remote homologs in the Protein Data Bank using HHblits/HHsearch. Since each ORFan cluster contained multiple non-redundant sequences, a non-trivial MSA and profile could be generated in each case. Thus, not only was the sequence clustering step useful in removing spurious ORFs, but it was also essential for generating the conservation profiles used in profile-profile comparison.

A considerable number of ORFans (73,896 sequences, 15.3%; 21,940 clusters, 15.8%) exhibited significant remote homology to proteins of known structure, with some metagenomes producing a greater fraction of annotated ORFans than others: 25.0% (GPC), 15.4% (GOS) and 9.3% (HG) of ORFan clusters (Table 1). This represents a new dataset of annotated, extremely divergent metagenome-derived proteins and provides a means to profile ORFan functions in general.

**Figure 2:**
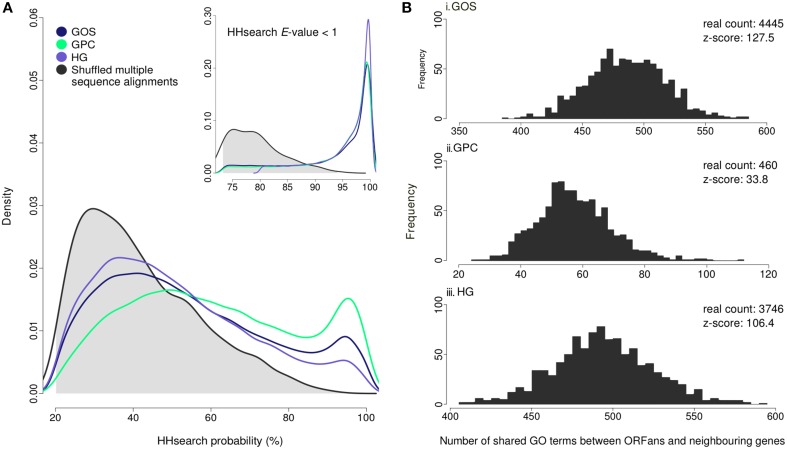
**Estimated false discovery rate of ORFan remote homology detection and functional prediction. (A)** Distributions of HHsearch probability scores for ORFans from three metagenomes, and shuffled sequences, searched against a PDB-derived HMM library. There is an abundance of high-scoring predictions (i.e., above 80% probability) for ORFan proteins compared to the expected (null) distribution. This separation becomes even greater when an HHsearch *E*-value threshold of 1 is applied (see inset). **(B)** The number of shared GO terms between functionally annotated ORFans (probability scores >80%) and their metagenomic neighbors (see Materials and Methods) is shown for three metagenomes. The null distributions, as estimated by randomly shuffling ORFan identities/positions, are shown along with the z-scores relative to these distributions. The mean values for the random distributions are: GOS (486.3), GPC (57.8), and HG (494.4).

Despite thorough benchmarking of HHblits/HHsearch (Remmert et al., 2011), there remains a possibility that the predictions are false positives due to factors associated with our pipeline and dataset. Therefore, we empirically measured a false discovery rate by repeating the entire procedure on an artificial dataset composed of ORFan clusters with shuffled sequences (Figure 2A). Specifically, 3000 random ORFan clusters were selected (1000 from each metagenome), and their alignment columns were shuffled, thereby preserving conservation information and compositional characteristics, while destroying potential similarity to real proteins. Any detectable homology between these artificial protein families and the PDB database indicates a false positive prediction. The random dataset generally produced low HHsearch probability scores, whereas the real metagenomic ORFans resulted in a large abundance of high-scoring predictions (Figure 2A). At a probability score of 80% or higher, the HHsearch method was able to annotate 15.8% of the real ORFan clusters and only 1.4% of false sequence clusters, which is indicative of a low (~9%) false discovery rate. This result provides support for the quality of the remote homology predictions, and suggests that many ORFans (15.3%) are divergent homologs of existing structural families.

### ORFan functions are consistent with those of their gene neighborhood

Given that a sizeable portion of metagenomic ORFans exhibit remote homology to protein structures, a key follow-up question concerns what functional information can be gained from these detected relationships. For functional annotation, we assigned the same GO terms as those associated with their identified remote PDB homologs. To assess whether the predicted ORFan functions are accurate and thus biologically meaningful, we measured their functional consistency with neighboring genes, a well established phenomenon in prokaryotes (Dandekar et al., 1998; Marcotte et al., 1999; Galperin and Koonin, 2000; Salgado et al., 2000; Yanai et al., 2002; Korbel et al., 2004). We reasoned that if predicted ORFan functions are accurate, they should show significantly elevated functional consistency compared to a random distribution (see Materials and Methods). Functional consistency was calculated as the number of shared GO terms between an ORFan and its metagenomic neighbors, defined as one gene on either side of an ORFan, in the same orientation and within a 1 kb boundary. As a statistical test, we computed the total number of shared GO terms for all annotated ORFans, and compared this to an estimated random distribution in which the ORFans were shuffled amongst their original locations. ORFans from all three metagenomes exhibited extremely high, statistically significant levels of functional consistency with their neighbors (Figure 2B). This effect was abolished completely when the ORFans randomly swap their positions. Overall, the significant functional congruence between ORFans and their gene neighbors suggests that the predicted functions are of high quality and thus potentially meaningful for biological interpretation.

### Enriched functions among ORFans

An important next question concerns the predicted ORFan functions themselves, how they compare to the homology-based functional profile inferred for the remaining metagenome, and what insights they may provide into hidden functions of their respective environments. To examine ORFan functions as a whole for each metagenome, we computed ORFan functional profiles as collections of GO terms and their frequencies, as based on previous studies (Tringe et al., 2005). We also calculated separate functional profiles for 10,000 Pfam-annotated CDSs of each metagenome as a reference, to which ORFan functions could be compared.

These comparisons reveal that ORFans possess a distinct functional profile from that of homology-annotatable proteins. This is evident from a clustering analysis in which the ORFan functional profiles from the three metagenomes group together (Figure S3). However, this is also somewhat expected since ORFans from different metagenomes will be inherently similar by virtue of *lacking* conserved functions present in the homology-annotated subset.

Consistent with the unique functional profile of ORFans, we identified numerous functions that were significantly overrepresented within the ORFans of each metagenome (Table 3, Table S1). These ORFan-enriched functions include terms relating to viral processes, carbohydrate metabolism, as well as several functions with particular relevance to their respective metagenomes (explored in following sections). We ensured that the reported functions are also significantly enriched (all with adjusted *p* < 0.05) compared to the reference database (PDB) and are thus not simply due to random matches to PDB entries.

**Table 3:** **Top five significantly enriched GO terms among ORFans in each metagenome relative to non-ORFans and the PDB**.

**GO term**	**ORFan clusters (individual sequences)**	**Proportion of ORFan clusters with GO term**	**Proportion of Pfam-annotated subset with GO term**	**Fold**	***p*-value against Pfam-annotated subset (adjusted)**	***p*-value against PDB70 (adjusted)**
**GPC**						
GDP-dissociation inhibitor activity	66 (157)	1.1 × 10^−2^	6.1 × 10^−4^	18.1	7.5 × 10^−55^	1.6 × 10^−90^
Dibenzothiophene catabolic process	35 (110)	5.9 × 10^−3^	4.9 × 10^−4^	12	1.7 × 10^−22^	3.7 × 10^−55^
Mitochondrial fission	28 (79)	4.7 × 10^−3^	3.7 × 10^−4^	12.8	2.2 × 10^−18^	7.1 × 10^−40^
Sequence-specific DNA binding	162 (415)	2.7 × 10^−2^	1.3 × 10^−2^	2.1	6.4 × 10^−14^	2.1 × 10^−49^
Viral release from host cell	14 (39)	2.3 × 10^−3^	1.2 × 10^−4^	19.2	1.2 × 10^−10^	5.1 × 10^−2^
**GOS**						
Polysaccharide catabolic process	62 (210)	7.2 × 10^−3^	4.5 × 10^−4^	16.3	1.2 × 10^−48^	8.0 × 10^−6^
L-ascorbic acid binding	89 (306)	1.0 × 10^−2^	1.8 × 10^−3^	5.8	4.7 × 10^−35^	1.0 × 10^−74^
ADP-heptose-lipopolysaccharide heptosyltransferase activity	35 (136)	4.1 × 10^−3^	2.2 × 10^−4^	18.4	1.6 × 10^−28^	4.0 × 10^−88^
Phosphatidylinositol alpha-mannosyltransferase activity	26 (104)	3.0 × 10^−3^	1.1 × 10^−4^	27.3	4.9 × 10^−25^	5.6 × 10^−42^
Endonuclease activity	157 (576)	1.8 × 10^−2^	6.7 × 10^−3^	2.7	1.6 × 10^−24^	1.2 × 10^−11^
**HG**						
Sequence-specific DNA binding	149 (617)	6.5 × 10^−2^	2.9 × 10^−2^	2.2	3.2 × 10^−15^	1.6 × 10^−94^
Polysaccharide catabolic process	49 (139)	2.1 × 10^−2^	4.6 × 10^−3^	4.6	1.3 × 10^−14^	1.2 × 10^−21^
Regulation of sporulation resulting in formation of a cellular spore	11 (74)	4.8 × 10^−3^	5.6 × 10^−4^	8.5	2.3 × 10^−4^	4.8 × 10^−20^
Ribonuclease activity	18 (88)	7.8 × 10^−3^	2.0 × 10^−3^	3.9	3.7 × 10^−3^	1.0 × 10^−3^

*Only four significantly enriched terms were identified for the HG metagenome*.

The detected enrichment of viral functions is consistent with previous suggestions that a large proportion of ORFans may be bacteriophage derived (Daubin and Ochman, 2004). Since viruses undergo rapid rates of evolution and are relatively undersampled in genomic databases, their proteins may also appear significantly divergent from database sequences. Our results provide strong support for this hypothesis since numerous virus-related functional terms are significantly enriched (adjusted *p* < 0.05) among the annotated ORFans (Table 3, Table S1). For example, the term “viral release from host cell” was among top enriched ORFan functions in the GOS (*p* = 1.1 × 10^−16^) and GPC metagenomes (*p* = 1.2 × 10^−10^). Other enriched functional terms associated with viruses include “RNA ligase” (Doherty et al., 1996), “lysozyme” (Fastrez, 1996), and “phospholipase” (Zádori et al., 2001) (Table 3, Table S1).

Although enriched, we estimate that viral sequences may be a relatively small proportion of ORFans overall, similar to previous reports (Yin and Fischer, 2006). That is, only 4.1% (GPC), 6.3% (GOS) and 5.6% (HG) of ORFans matched viral protein structures (Table S2), while the majority matched structures of bacterial origin. Interestingly, however, the proportions of viral PDB matches are roughly four-fold higher than that observed for the homology-annotatable proteins which ranges from 1.4 to 2.4%, which provides additional support for an enrichment of viral functions among metagenomic ORFans.

Another common function overrepresented in the ORFans of all three metagenomes relates to carbohydrate degradation or transport. This finding is consistent with the considerable sequence and structural diversity of carbohydrate-active enzymes (Cantarel et al., 2009). Enriched carbohydrate-related functions among ORFans include “polysaccharide catabolic process” in all three metagenomes (all with *p* < 1 × 10^−5^), “cellulase activity” (*p* = 6.1 × 10^−7^) in the GPC metagenome and “phosphatidylinositol alpha-mannosyltransferase activity” in the GOS metagenome (*p* = 4.9 × 10^−25^) (Table 3, Table S1).

Ultimately, both the clustering and enrichment analyses demonstrate that ORFan functions do not merely mirror the functions expected from homology-annotatable proteins. Thus, the efforts of remote homology detection have uncovered a highly divergent sequence space, including viral proteins and carbohydrate-active enzymes, which was not detectable in the annotatable subset of each metagenome.

### Environment-specific ORFan families and functions

Potentially more interesting than the functions generally enriched among ORFans are the specific ORFan families and functions unique to each environment. Indeed, it has been hypothesized that ORFans may be unique in their potential to encode ecologically important functions (Wilson et al., 2005). One explanation for this is that environment-specific functions may be encoded in part by environment-specific genes that differ from characterized genes in the database.

**Figure 3:**
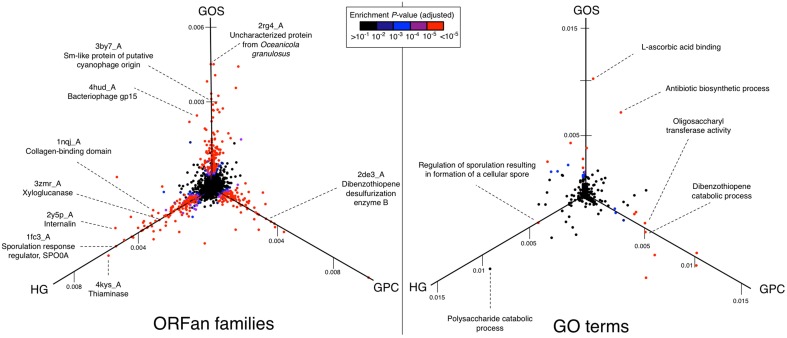
**Metagenome-specific ORFan families and functions**. Shown are projections of three-dimensional scatterplots in which each axis indicates the proportion of ORFans from a specific metagenome with a specific annotation (left panel—families; right panel—functions). ORFan families are defined based on their top remote homology match in the PDB database, and functions are defined by GO terms as described in the Methods. Data points that project uniquely along one axis therefore indicate metagenome-specific ORFan families or functions, while those close to the origin indicate similar proportions among all three metagenomes. Cases described in the text have been labeled.

To explore this in greater detail, we visualized metagenome-specific ORFan functions using 3D scatterplots (Figure 3), similar to previous three-way comparisons of metagenome functional profiles (Tringe et al., 2005). In these plots, ORFan functions that are of similar abundance in all three metagenomes will appear close to the origin, whereas ORFan functions that are relatively abundant in one metagenome will project outwards along that metagenome's axis. In addition to GO terms, we also performed the same analysis at the level of ORFan families, as represented by the top identified remote homolog in the PDB.

This three-way comparison reveals several broad functions (Figure 3, right) and a much larger number of families (Figure 3, left) that are significantly enriched in the ORFans from one metagenome. Below we highlight some interesting examples.

#### HG-specific ORFans

Several of the most abundant HG-specific ORFan families have predicted roles involved in gut metabolism and host interactions. These include HG-specific ORFan homologs of thiaminase, an enzyme that breaks down vitamin B1, the virulence factor internalin, and the collagen-binding domain which could play roles in gut adherence or invasion (Figure 3).

Most intriguing are the ORFans with predicted functions in “polysaccharide catabolic process,” a function that is significantly enriched (*p* = 1.3 × 10^−14^, Table 3) in the HG metagenome (Figure 3). This is of great interest in the context of the human gut microbiome because breakdown of indigestible dietary polysaccharides is one of the fundamental roles of intestinal bacteria (Flint et al., 2008). Among the most abundant HG-specific ORFan families is one with detected remote homology to PDB ID 3zmr, a crystal structure of xyloglucanase from the common human gut organism, *Bacteroidetes* (Larsbrink et al., 2014). This enzyme functions in the gut microbial digestion of the plant-cell wall derived polysaccharide, xyloglucan (XyG), and was only recently characterized as the first xyloglucanase enzyme in the gut microbial community (Larsbrink et al., 2014). The HG-specific ORFans identified here exhibit remote homology to the Bacteriodetes-Associated Carbohydrate-binding Often N-terminal (BACON) domain within these enzymes, suggesting a function in gut carbohydrate metabolism.

Another HG-specific ORFan family includes 74 ORFan proteins from 11 sequence clusters in the HG metagenome with a predicted function in regulation of sporulation. This was the third most enriched function (by fold) among HG ORFans (*p* = 2.3 × 10^−4^, Table 3) and yet was not enriched in the other two metagenomes as illustrated in Figure 3. These ORFans are primarily distant homologs of the DUF199/WHIA transcriptional regulator or the sporulation response regulator, SPO0A. While sporulation is a general function also observed elsewhere, numerous studies have demonstrated its particular enrichment within the human gut microbiome. This has been attributed to the relative abundance of gut Firmicutes species, which include many spore-forming members (Turnbaugh et al., 2007). However, specific genes and sporulation pathways may be unique to the human gut microbiome. For instance, a recent analysis of *Lachnospiraceae* genomes revealed that key sporulation-related genes are exclusive to human gut associated *Lachnospiraceae* and absent elsewhere (Meehan and Beiko, 2014). It is therefore interesting that both ORFans and homology-annotatable proteins from the gut microbiome show this functional pattern. This data further implicates sporulation as a particularly important function within the human gut community, and provides motivation for further exploration of divergent gut sporulation proteins.

#### GOS-specific ORFans

Several abundant GOS-specific ORFan families and functions are indicated in Figure 3. Enriched functions include antibiotic biosynthesis and L-ascorbic acid (vitamin C) binding. Interestingly, the most abundant GOS-specific ORFan families show patterns consistent with a marine environment. These include a family of ORFans with remote homology to a cyanophage (an abundant marine virus that infects oceanic cyanobacteria) protein, and another family with remote homology to PDB ID 2rg4, an uncharacterized protein from the marine bacterium, *Oceanicola granulosus*. The identification of GOS-specific ORFans matching viral structures (see Figure 3 for another example, bacteriophage gp15) is consistent with Yooseph et al. (2007) who reported a viral origin for a significant number of divergent GOS sequences.

#### GPC-specific ORFans

One of the most interesting GPC-specific ORFan families has remote homology to dibenzothiophene (DBT) desulfurization enzyme B (PDB ID 2de3_A). This is also a significantly enriched ORFan function compared to non-ORFans from the same metagenome (*p* = 1.7 × 10^−22^, Table 3). DBT desulfurization genes have been identified in petroleum-polluted soils where they are implicated in DBT degradation, and are of interest to the oil industry to reduce the levels of sulfur in fuel (Duarte et al., 2001).

### Targeted discovery of ORFan metalloproteases

Regardless of whether a particular function is overrepresented among ORFans and/or metagenome-specific, its detection within ORFans may be valuable for its own sake to expand its knowledge and sequence space. Indeed, metagenomes are a useful resource for the discovery of novel families of biotechnologically and scientifically important enzymes such as glycosyl hydrolases (Li et al., 2009) and proteases (Waschkowitz et al., 2009).

**Figure 4:**
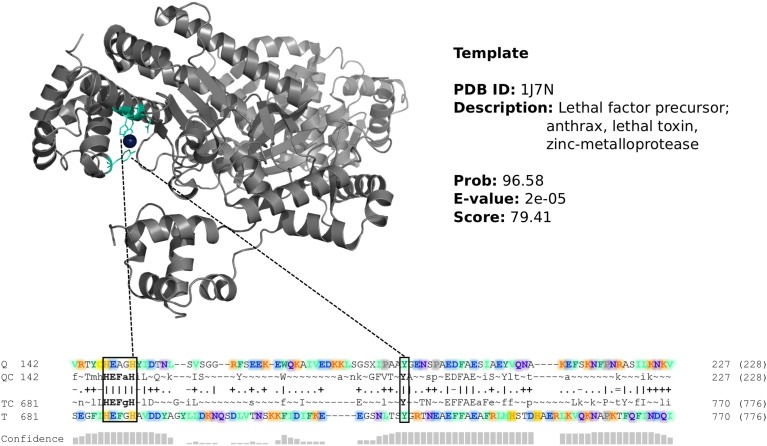
**One example of 257 predicted metalloprotease ORFan sequence clusters**. The example shown is a predicted metalloprotease ORFan from the HG metagenome with similarity to the protease domain of the anthrax toxin. The catalytic zinc-metalloprotease (HExxH) catalytic motif is conserved between the query and template, however the remaining sequence similarity is weak. In general, ORFan metalloproteases were predicted based on detected remote homology to protein structures of known or putative proteases and peptidases, as well as presence of the HExxH motif.

To explore its potential as a resource for enzyme discovery, we mined the annotated ORFans for novel metalloproteases. Metalloproteases are of particular biological (Nagase and Woessner, 1999; Duarte et al., 2014), evolutionary (Rawlings and Barrett, 1995; Doxey et al., 2008; Mansfield et al., 2015) and biotechnological (Adekoya and Sylte, 2009) interest. “Metallopeptidase activity” was also a significantly enriched function among ORFans from the GOS metagenome (*p* = 1.6 × 10^−20^, Table S1). Lastly, we also selected metalloproteases as a target function because these enzymes possess a convenient functional motif that provides additional evidence of predicted activity; namely, a conserved, zinc-binding, catalytic motif (HExxH). Remarkably, we identified 257 ORFan sequence clusters possessing both this motif and significant remote homology to protease or peptidase structures (Table 4). One example is highlighted in Figure 4, in which a predicted ORFan family from the HG displays significant remote homology to the zinc-metalloprotease domain of the anthrax toxin. Although the overall sequence similarity is quite weak, there are short regions of motif similarity and numerous residues within the catalytic site are conserved. The 257 ORFan subfamilies represent a rich resource of highly divergent metalloproteases that await future experimental characterization.

**Table 4:** **Predicted ORFan clusters with the HExxH motif and remote homology to metalloprotease structures**.

	**Number of clusters**	**Remote homology match (PDB entry and description)**
GPC	96	Total
	10	3cqb_A Peptidase M48
	8	4jix_A Peptidase M56
	8	4in9_A Peptidase M10, Matrixin
GOS	132	Total
	24	3cqb_A Peptidase M48
	11	4jiu_A DUF45 metallopeptidase
	10	4jix_A Peptidase M56
HG	29	Total
	5	3dte_A DUF955 peptidase-like domain
	3	3b4r_A Peptidase M50
	3	2y6d_A Peptidase M10

*The top three most abundant clusters by PDB match are listed*.

## Discussion

We developed a pipeline to identify and structurally annotate ORFans from three large and highly distinct metagenomes. Our results demonstrate that a considerable fraction (15.3%) of metagenomic ORFans exhibit remote but significant homology to structurally characterized proteins. This is surprising since neither BLAST nor profile-based methods were able to annotate them. These findings are consistent with previous structural studies that have consistently revealed ORFans to be divergent members of existing protein families (Godzik, 2011). For instance, a previous analysis of 248 structures of domains of unknown function (DUF) families selected from Pfam, determined that ~2/3 are divergent members of known protein families (Jaroszewski et al., 2009). These structural studies, together with the 15.3% of annotated ORFans presented here, support a classic duplication-divergence model (Ohno, 1970) in which ORFan genes might arise when one of two duplicated genes (paralogs) diverge rapidly to a point where homology becomes undetectable.

While initially attributed to an inadequate knowledge of sequence space, pseudogenes or prokaryotic “junk DNA” (Andersson and Andersson, 2001; Mira et al., 2002), or incorrectly annotated genes (Schmid and Aquadro, 2001), there is considerable evidence that many detected ORFans are functional (Hu et al., 2009). A functional role for many ORFans is also supported by the many high quality functional annotations we were able to predict. These annotations are themselves supported by a low estimated false discovery rate based on non-homologous shuffled sequences, as well as the significant level of functional similarity detected between ORFans and their neighboring genes.

The overrepresented functions among ORFans are also consistent with previous but debated (Yin and Fischer, 2006) claims that ORFans tend to be of viral and other mobilomic origins (Doherty et al., 1996; Cortez et al., 2009). For instance, one study examined 119 prokaryotic genomes for gene clusters exhibiting atypical sequence composition and found that over 39% of ORFans were contained within these clusters, strongly suggesting that integrative elements are a major evolutionary source of ORFans (Cortez et al., 2009). Viral and mobilomic origins of ORFans make sense from a biological perspective given the rapid mutation rates observed in viral DNA as well as a technical one given the relative undersampling of viral sequences in the database.

Lastly, our results agree with previous suggestions that ORFans encode environment-specific roles (Kaessmann, 2010; Tautz and Domazet-Lošo, 2011), specifically through the many metagenome-specific ORFan families and functions that we identified (Figure 3). Indeed, ORFans have been implicated in taxon-specific functions (Wilson et al., 2005) and lineage-specific developmental or morphological adaptations (Kaessmann, 2010; Tautz and Domazet-Lošo, 2011; Böttger et al., 2012).

Although annotatable ORFans may represent a relatively minor component of a metagenome, they differ dramatically in their functional profiles from typical, homology-annotatable proteins. Their inclusion within metagenome annotation pipelines may not significantly alter overall estimates of metagenome functional profiles, but they are themselves interesting to pursue and expand our understanding of key protein functions of interest. Ultimately, ORFan characterization through remote homology provides a glimpse into the highly divergent, occasionally viral, and environmentally important functions they contribute to their respective microbial communities.

## Resource

All ORFan predictions are available at http://doxey.uwaterloo.ca/ORFans/. The resource contains predicted ORFan protein sequences in FASTA format for each metagenome, as well as data files that include predicted ORFan relationships to PDB structures, functional descriptions, and additional statistics.

### Conflict of interest statement

The authors declare that the research was conducted in the absence of any commercial or financial relationships that could be construed as a potential conflict of interest.

## Supplementary material

Figure S1 **ORF length (# nucleotides) distributions for homology-annotatable vs. ORFan sequences from three metagenomes**. The relative abundance of ORFans decreases with increasing read length, which reflects the tendency for ORFans to be shorter than average proteins.

Figure S2 **GC content distributions for homology-annotatable vs. ORFan sequences from three metagenomes**. Homology-annotatable vs. ORFan sequences display highly similar GC content distributions within the same environment, but these distributions differ significantly between environments.

Figure S3 **Heatmap of GO function terms in the Pfam-annotated subset and the ORFan subset**. Only terms enriched (>1.25 fold) in at least one dataset are included in the heatmap to avoid display of invariant functions.
