# Supplementary figures and images for: Remote homology and the functions of metagenomic dark matter
Source: Front Genet. 2015 Jul 21;6:234. doi: 10.3389/fgene.2015.00234 (PMC4508852; doi:10.3389/fgene.2015.00234)

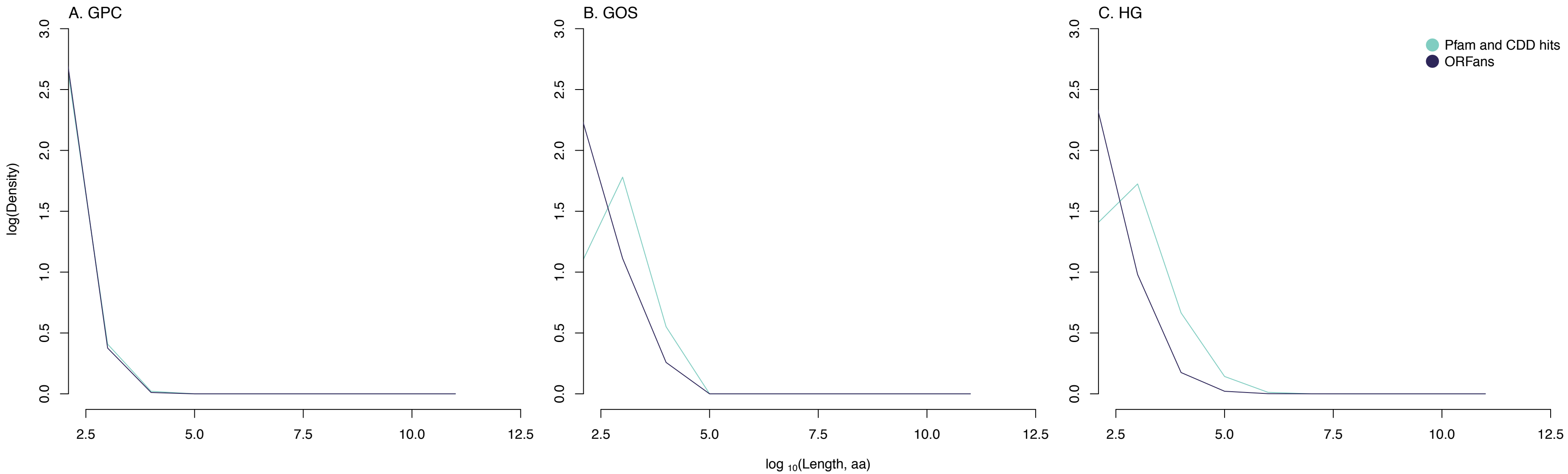

Supplement: Figure S1 — ORF length (# nucleotides) distributions for homology-annotatable vs. ORFan sequences from three metagenomes. The relative abundance of ORFans decreases with increasing read length, which reflects the tendency for ORFans to be shorter than average proteins. [file Image1.PDF]

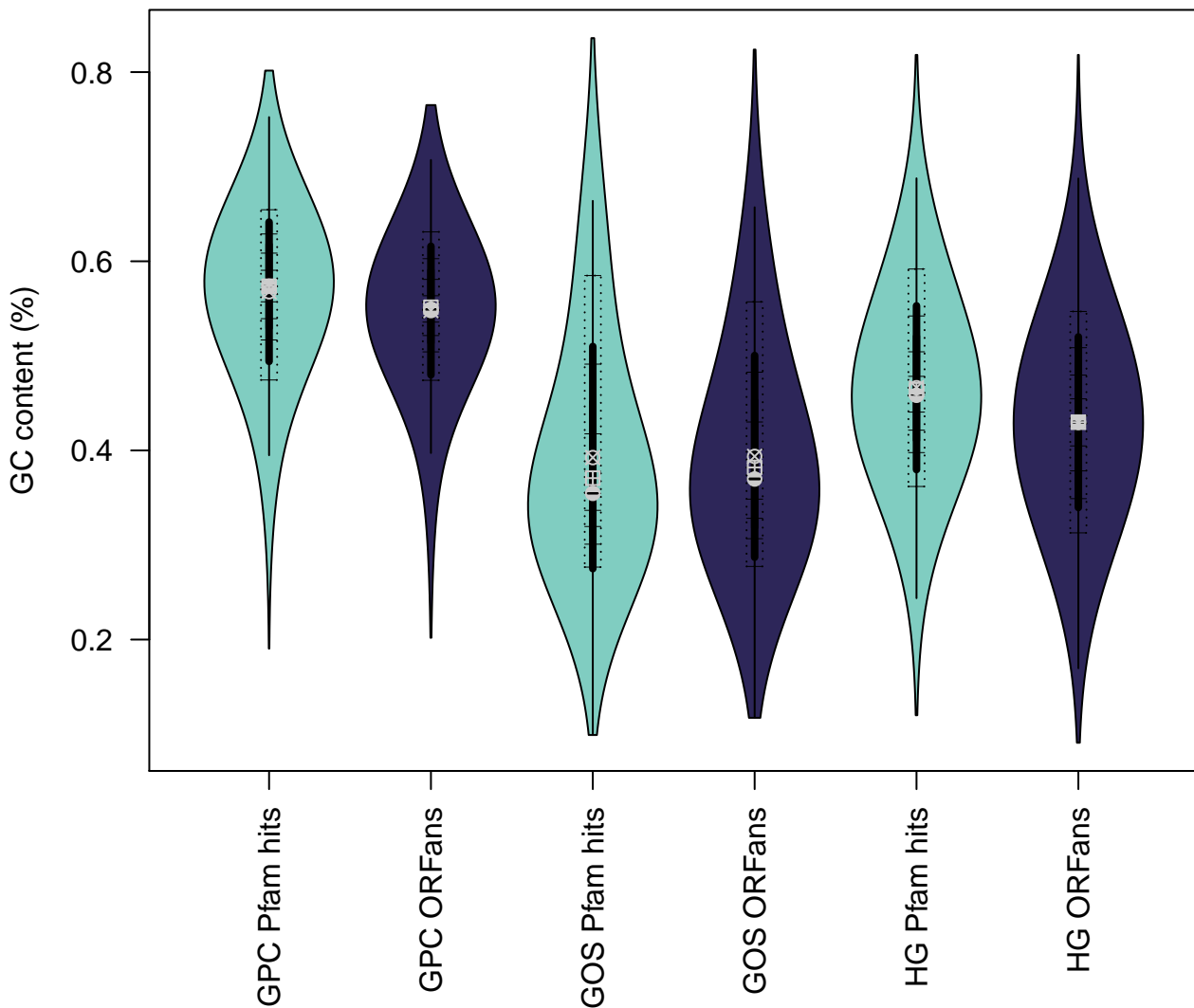

Supplement: Figure S2 — GC content distributions for homology-annotatable vs. ORFan sequences from three metagenomes. Homology-annotatable vs. ORFan sequences display highly similar GC content distributions within the same environment, but these distributions differ significantly between environments. [file Image2.PDF]

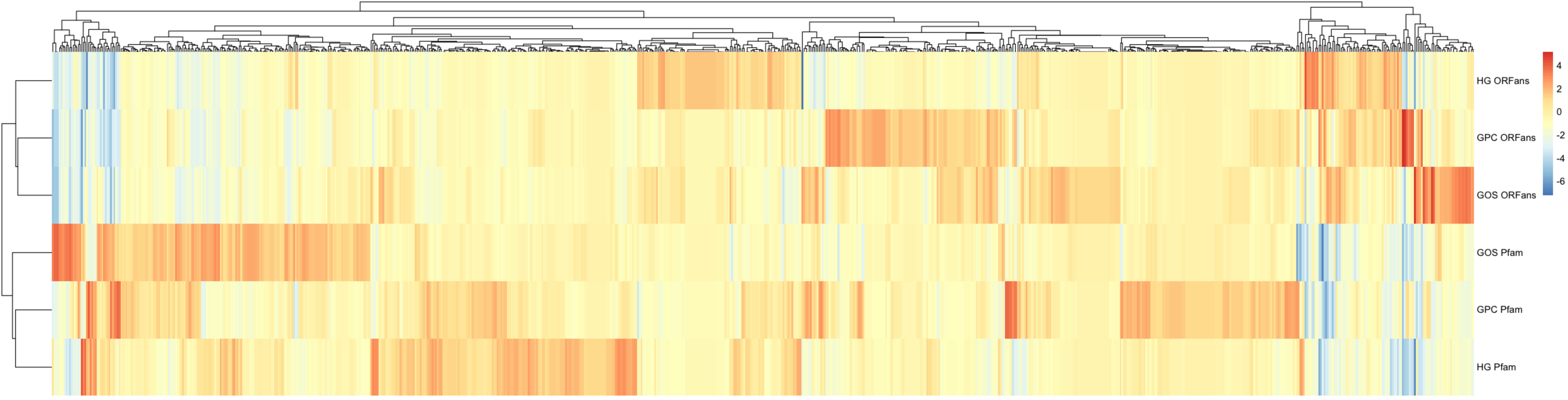

Supplement: Figure S3 — Heatmap of GO function terms in the Pfam-annotated subset and the ORFan subset. Only terms enriched (>1.25 fold) in at least one dataset are included in the heatmap to avoid display of invariant functions. [file Image3.PDF]
