# Supplementary material for: Remote homology and the functions of metagenomic dark matter
Source: Front Genet. 2015 Jul 21;6:234. doi: 10.3389/fgene.2015.00234 (PMC4508852; doi:10.3389/fgene.2015.00234)
Supplement: Supplementary file 5 [file Table2.DOCX]

**Table S2.** Taxonomic composition of remote PDB matches to ORFans versus homology-annotatable proteins from three large metagenomes.

Homology-annotatable proteins

|  | GPC (%) | GOS (%) | HG (%) |
| --- | --- | --- | --- |
| Virus | 1.4 | 1.4 | 2.4 |
| Bacteria | 75.5 | 75.0 | 80.5 |
| Archaea | 7.4 | 7.2 | 7.8 |
| Eukaryota | 15.7 | 16.5 | 9.2 |

ORFans

|  | GPC (%) | GOS (%) | HG (%) |
| --- | --- | --- | --- |
| Virus | 4.9 | 7.6 | 8.9 |
| Bacteria | 65.1 | 58.3 | 56.6 |
| Archaea | 6.7 | 6.6 | 7.1 |
| Eukaryota | 23.3 | 27.4 | 27.5 |
